# BAG Family Members as Mitophagy Regulators in Mammals

**DOI:** 10.3390/cells11040681

**Published:** 2022-02-15

**Authors:** Sophie Pattingre, Andrei Turtoi

**Affiliations:** Tumor Microenvironment and Resistance to Treatment Laboratory, Institut de Recherche en Cancérologie de Montpellier (IRCM), INSERM U1194, Université de Montpellier, Institut régional du Cancer de Montpellier, 34298 Montpellier, France; andrei.turtoi@inserm.fr

**Keywords:** autophagy, mitophagy, quality control, BAG

## Abstract

The BCL-2-associated athanogene (BAG) family is a multifunctional group of co-chaperones that are evolutionarily conserved from yeast to mammals. In addition to their common BAG domain, these proteins contain, in their sequences, many specific domains/motifs required for their various functions in cellular quality control, such as autophagy, apoptosis, and proteasomal degradation of misfolded proteins. The BAG family includes six members (BAG1 to BAG6). Recent studies reported their roles in autophagy and/or mitophagy through interaction with the autophagic machinery (LC3, Beclin 1, P62) or with the PINK1/Parkin signaling pathway. This review describes the mechanisms underlying BAG family member functions in autophagy and mitophagy and the consequences in physiopathology.

## 1. Introduction

The maintenance of cellular homeostasis depends on the tight equilibrium between anabolism and catabolism. Two catabolic pathways ensure the degradation of intracellular material: the ubiquitin–proteasome system and autophagy, a cell digestion process that ends in the lysosome. For their response to stress, cells have developed three autophagic processes: (i) chaperone-mediated autophagy that involves heat shock cognate protein 70 (HSC70) and lysosome-associated membrane glycoprotein 2a (LAMP2a) to specifically degrade proteins with a KFERQ sequence; (ii) microautophagy, in which invaginations of the lysosomal membrane allow the sequestration of a small portion of the cytoplasm; and (iii) macroautophagy.

**Figure 1:**
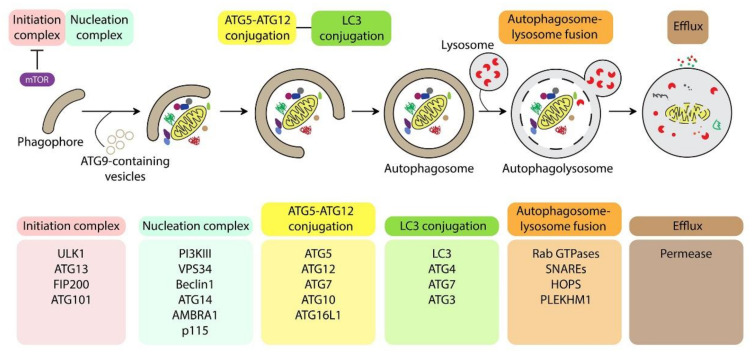
Autophagosome formation, maturation, and degradation require the autophagic core machinery. Autophagy is a multistep process that begins with the nucleation of a double membrane called a phagophore. The ULK1 complex and the nucleation complex ensure autophagy initiation, whereas ATG9 vesicles allow the shuttling of membrane sources. The expansion of autophagosomal membranes is dependent upon the two conjugation systems and allows the sequestration of intracytoplasmic material. The degradative properties are acquired after the fusion between autophagosome and lysosome. After completion, the degraded material is recycled into the cytoplasm via permeases [5,6].

Macroautophagy, called hereafter autophagy, is a major lysosomal catabolic pathway for the degradation and recycling of intracellular materials, such as lipids, proteins, nucleic acids, and organelles. Depending on the condition, the materials are randomly or selectively sequestered into a double membrane vacuole, called an autophagosome. Then, autophagosomes undergo a maturation process by fusion with the lysosome for degradation [1]. The autophagic process is regulated by more than thirty autophagy-related (ATG) proteins [2]. In concert with the nucleation of the phagophore, the membrane transporter complex with ATG9-containing vesicles provides membrane sources. Next, six functional groups are involved in the autophagic process: (1) the initiation complex, which requires the inhibition of the kinase mTOR and contains the ULK1 (ATG1) kinase and ATG13, among others; (2) the nucleation complex with phosphatidylintol 3-kinase class III (PI3KIII) and Beclin1 (ATG6); (3) the ATG12-ATG5-ATG16L elongation complex; (4) the protein light chain 3 (LC3) family/phosphatidylethanolamine elongation/conjugation system [3]; (5) the autophagosome/lysosome fusion complex composed of Rab GTPases, soluble NSF (N-ethylmaleimide-sensitive factor) attachment protein receptor (SNARE), homotypic fusion and protein sorting (HOPS), and Pleckstrin homology domain-containing family member 1 (PLEKHM1); and (6) the efflux machinery to allow the recycling of nutrients (Figure 1).

LC3, the mammalian homolog of *ATG8*, is used as a marker of autophagy. Indeed, after synthesis, pro-LC3 is first cleaved into LC3-I which is located in the cytoplasm. Upon autophagy induction, LC3-I matures into LC3-II, which binds to the autophagosomal membrane through a covalent interaction with phosphatidylethanolamine. LC3-II maturation can be quantified by western blotting (electrophoretic shift from 18 to 16 KDa) or by fluorescence analysis (cytoplasmic staining for LC3-I and punctuated structures representing autophagomes for LC3-II) [4].

**Figure 2:**
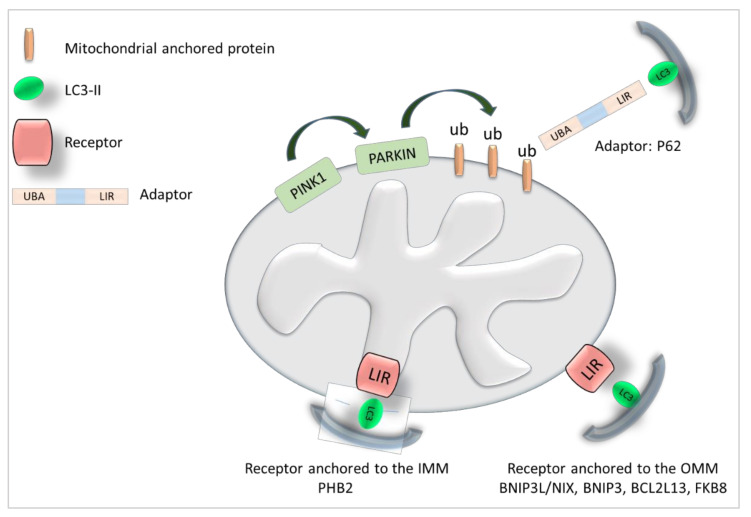
The specific targeting of mitochondria to autophagosomes is mediated by adaptors and receptors. Mitophagy specificity is ensured by mitophagic receptors anchored to the OMM (BNIP3L/NIX [8], BNIP [9], P3BCL2L13 [10], PHB2 [11] and FKBP8 [12]) or to the inner mitochondrial membrane (IMM) and by cytoplasmic adaptors, such as P62 [13]. Mitophagy induction involves the kinase PINK1 and the E3 ubiquitin-protein ligase Parkin. After mitochondrial depolarization, PINK1 accumulates at the OMM and activates Parkin by phosphorylation, allowing its mitochondrial recruitment. Parkin ubiquitinates OMM proteins [14] that are recognized by adaptors, such as P62/sequestosome 1, for autophagic elimination [15]. LC3-II interacts with the LIR motif of adaptors and receptors.

Autophagic cargos may also be delivered to the autophagosome in a selective manner. Thus, selective autophagy enables the specific targeting of intracellular materials to autophagosomes. For example, the degradation of cellular aggregates through autophagy is named aggrephagy. Pexophagy allows the degradation of peroxisomes, and xenophagy targets intracellular pathogens to autophagomes. Moreover, the degradation of ubiquitylated misfolded proteins by autophagy, which occurs mainly in brain and muscle, is called chaperone-assisted selective autophagy (CASA) [7]. The selective targeting of organelles to autophagosomes is mediated by receptors or adaptors that bind to the autophagosomal-bound form of LC3 (LC3-II). These receptors/adaptors harbor a LC3-interacting region (LIR) that is defined by the W/F/YxxL/I sequence and is essential for the interaction with LC3-II [8] (Figure 2).

One of the most studied form of selective autophagy is the degradation of mitochondria through mitophagy [9] (Figure 2). Mitochondria ensure oxidative phosphorylation reactions thanks to the maintenance of a gradient across the IMM and the mitochondrial matrix. The loss of this proton gradient leads to mitochondrial depolarization. Then, to ensure the specificity of the degradation of altered/depolarized mitochondria, specific mitophagic receptors anchored to the mitochondrial membrane link mitochondria and autophagosomes. In mammalian cells, several mitophagic receptors have been identified, such as BCL2-adenovirus E1B 19 kDa protein-interacting protein 3-like/ Nip3-like protein X (BNIP3L/NIX) [10], BCL2-adenovirus E1B 19 kDa protein-interacting protein 3 (BNIP3) [11], BCL2 Like 13 (BCL2L13) [12], prohibitin 2 (PHB2) [13], and FK506-binding protein (FKBP8) [14]. The specific degradation of mitochondria is also ensured by cytoplasmic adaptors, such a P62 (also called sequestosome 1), that target the autophagosome via LC3 and the mitochondria via specific signaling events [15].

The most characterized signaling pathway implicated in mitophagy induction involves PTEN-induced putative kinase 1 (PINK1) and the E3 ubiquitin-protein ligase Parkin. After mitochondrial depolarization, PINK1 accumulates at the outer mitochondrial membrane (OMM) and activates Parkin by phosphorylation, allowing its mitochondrial recruitment. Once activated, Parkin ubiquitinates OMM proteins, and then PINK1 phosphorylates ubiquitin residues on serine 65 [16]. Ubiquitinylated OMM proteins are recognized by specific adaptors, such as P62, allowing the engulfment of mitochondria into autophagosomes for autophagic elimination [17] (Figure 2). Interestingly, the PINK1/Parkin pathway may also play a role in receptor-dependent mitophagy.

If autophagy represents one of the main mechanisms for the maintenance of cellular homeostasis, another critical point of control concerns protein quality control, also called proteostasis. This mechanism requires the triage of misfolded proteins that will undergo refolding or degradation through the proteasome or the chaperone-mediated autophagy pathway to avoid protein aggregation, for example. Chaperones, by their ability to recognize misfolded proteins, are essential in this process. In stress conditions that may affect cell functions, heat shock proteins (HSPs) are the most important family of molecular chaperones. The proteostasis machinery involves also co-chaperones that directly interact with chaperones, thus modifying their activity or interactome [18].

The BCL-2-associated athanogene (BAG) family is a multifunctional group of co-chaperones that are evolutionarily conserved from yeast to mammals. The BAG domain is required for their interaction with the ATPase domain of HSP/HSC70, acting as a nucleotide exchange factor. The objective of this review is to summarize recent studies that highlight the role of BAG family members in autophagy and mitophagy.

## 2. BAG Family Members in the Regulation of Autophagy and Selective Autophagy

**Figure 3:**
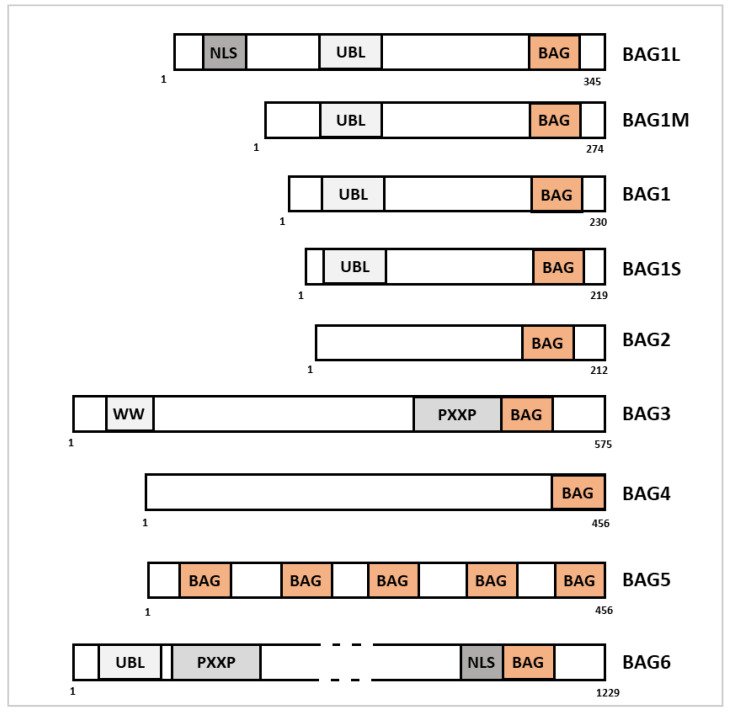
Sequence alignment of the human BAG proteins. The BAG family has six members (BAG1 to BAG6). Each family member contains a conserved BAG domain (orange) in its C-terminal region, except BAG5, which has five BAG domains. Four BAG1 isoforms, generated by alternative splicing, have been identified (BAG1L, BAG1M, BAG1, BAG1S). In addition to the BAG domain, BAG family members contain specific domains that are mainly involved in protein–protein interaction (UBL, PXXP). BAG1L and BAG6 also contain a nuclear localization signal (NLS) that allows their nucleo-cytoplasmic shuttling.

The sequences of the six BAG family members include many specific domains implicated in various cell quality control functions, such as autophagy, apoptosis, and proteasomal degradation of misfolded proteins. They all have a BAG domain in the C-terminal region that is composed of 110–130 amino acids and forms three alpha helices of 30–40 amino acids. The BAG domain allows the interaction with the ATPase domain of the HSC70/HSP70 chaperones [19]. Each BAG family member contains one BAG domain, with the exception of BAG5, which has five BAG domains (Figure 3). Recently, the role of BAG family members has been highlighted (Table 1).

### 2.1. BAG1

BAG1 was discovered due to its anti-apoptotic function in a screen using BCL2 as bait [20]. There are four isoforms generated by alternative splicing [21] that contain a ubiquitin-like (UBL) domain and the BAG domain (Figure 3).

BAG1 stimulates autophagy during cardiac adaptation, which is essential for heart protection after ischemia/reperfusion injury. During ischemic adaptation, LC3-II, Beclin1, and BAG1 are upregulated. In addition, BAG1 interacts (co-immunoprecipitation experiments) and co-localizes with LC3-II. However, the role and mechanism of this interaction remain unknown. Furthermore, BAG1 silencing in vivo (rat myocardium) and in vitro (myoblasts) decreases cardiac adaptation after ischemia/reperfusion injury, LC3-II and Beclin1 expression, and autophagy [22].

Beside the interaction with LC3, the BAG1S and BAG1L isoforms interact with Beclin1 in breast cancer cell lines. Since the intracellular localization of BAG1 variants differs, the authors analyzed the co-localization between BAG1 variants and Beclin1 and observed that only BAG1S co-localizes with Beclin1, suggesting that BAG1S/Beclin1 interaction may be physiologically relevant compared to BAG1L [23].

During aging, the expression levels of BAG1 and BAG3 are inversely correlated, allowing a switch between proteasomal degradation and autophagy (see below, Section 3.3).

### 2.2. BAG2

BAG2 is expressed in many tissues and in various cell organelles, such as mitochondria, endoplasmic reticulum (ER), and microtubules. Growing evidence indicates that BAG2 is involved in diseases such as cancer and neurodegenerative disorders [24].

To date, BAG2’s role in autophagy and selective autophagy remains largely unknown. It has been reported that BAG2 promotes macrophage survival after *Mycobacterium tuberculosis* infection by limiting ER stress through the induction of reticulophagy. The authors showed that BAG2, which has no LIR motif, interacts with P62, allowing the specific targeting of ER to autophagomes. BAG2 also stimulates autophagy by disrupting Beclin1/BCL2 interaction [25]. However, in another study, BAG2 silencing in breast cancer cells induced apoptosis but did not affect LC3B protein levels [26]. These seemingly contrasting findings suggest that more studies are necessary to fully elucidate BAG2’s role in autophagy.

### 2.3. BAG3

BAG3, probably the most studied BAG family member, plays a role in neurodegenerative diseases, viral infections, cardiomyopathy, and cancer [27]. One of the main functions of BAG3 is the maintenance of proteostasis in stressed and aged cells through the regulation of selective autophagy. Indeed, BAG3 promotes chaperone activity, favors the formation of aggresomes, and enhances CASA [28]. CASA is induced by the association between BAG3, HSPB8, HSP70, and the protein targeted for degradation. Once this complex is formed, the E3 ubiquitin ligase CHIP poly-ubiquitinates the target protein that then interacts with P62, allowing its engulfment in autophagosomes and degradation [29].

The CASA complex plays a crucial role in the degradation of protein aggregates implicated in neurodegenerative diseases, such as mutated huntingtin [30], mutated superoxide dismutase 1 in the familial form of amyotrophic lateral sclerosis [31], and tau in Alzheimer’s disease [32].

Interestingly, BAG1 and BAG3 compete for the degradation of poly-ubiquitinylated proteins. BAG1 is involved in their proteasomal degradation, whereas BAG3 modulates autophagy via the CASA complex. In aging, a switch in the expression of these proteolytic systems occurs in favor of BAG3, thus explaining why autophagy is the privileged catabolic process in aged cells [33]. Similarly, a switch from BAG1 to BAG3 occurs in Duchenne muscular dystrophy to route damaged proteins towards degradation by autophagy [34].

BAG3 is also implicated in cardiomyopathies. *Bag3*^−/−^ mice and mice harboring a mutation in BAG3 (BAG3^P209L^) develop severe cardiomyopathy. In striated muscle, the BAG3–CASA complex is localized in the Z-disk of sarcomeres [35], and BAG3 knock-down leads to protein aggregate accumulation in cardiomyocytes [36].

In cancer, BAG3-dependent autophagy is strongly associated with drug resistance, and in many cancer types, such as colon and pancreatic cancer, high BAG3 expression is a poor prognostic factor [37]. BAG3 plays a role in cancer also by modulating cell metabolism [38].

### 2.4. BAG4

The role of BAG4 in autophagy, mitophagy, or other selective forms of autophagy is unknown.

### 2.5. BAG5

BAG5 is unique among the BAG family members, because it contains five BAG domains. In hepatocellular carcinoma, BAG5 promotes autophagy after treatment with sorafenib, a kinase inhibitor, conferring drug resistance [39].

BAG5 is also implicated in selective autophagy through its interaction with P62 in the context of Parkinson’s disease (PD). Indeed, BAG5 knock-down reduces P62 protein levels and promotes the formation of alpha-synuclein oligomers. Although the molecular mechanism underlying this interaction remains to be elucidated, BAG5 may promote aggrephagy, which plays a role in PD pathogenesis [40].

### 2.6. BAG6

The first evidence of BAG6’s role in autophagy came from the observation that it can modulate the activity of the acetyltransferase EP300 [40,41]. Indeed, BAG6 regulates autophagy by acting as a nucleocytoplasmic vehicle for EP300, thus controlling its localization and accessibility to nuclear (P53) and cytoplasmic (ATG proteins) substrates involved in autophagy [42,43]. When BAG6 and EP300 are in the cytoplasm, ATG proteins are acetylated by EP300, and autophagy is inhibited. Conversely, when they are in the nucleus (e.g., after starvation), ATG protein acetylation is decreased and EP300-dependent acetylation of p53 promotes the expression of pro-autophagic genes and autophagy.

During ER stress, BAG6 is cleaved by caspase 3, leading to its cytoplasmic localization and its interaction with pro-LC3 and LC3-I via the LIR motif (LIR^132−135^). In this case, BAG6 sequesters LC3-I, preventing autophagosome formation and promoting apoptosis [44].

**Table 1:** Role of BAG family members in autophagy regulation.

Bag Family Member	Role in Autophagy
**BAG1**	Stimulates autophagy during cardiac adaptation after ischemia/reperfusion injury [22]: - Interaction with LC3-II - Co-localization with LC3 In breast cancer cell lines [23]: - BAG1S and BAG1L interact with Beclin1 - BAG1S co-localizes with Beclin1
**BAG2**	Induction of reticulophagy after *mycotuberculosis* infection [25]: - Interaction with P62 - Disruption of Beclin1/BCL2 complex
**BAG3**	Promotion of chaperone-assisted selective autophagy [28] Stimulation of autophagy leading to drug resistance in colon and pancreatic cancer [37]
**BAG5**	Stimulation of autophagy during sorafenib treatment in hepatocellular carcinoma leading to drug resistance [30] Promotion of aggrephagy through interaction with P62 in PD [40]
**BAG6**	Modulation of autophagy in function of its intracellular localization [42,43]: - In the cytoplasm at basal level: BAG6 sequesters EP300 in the cytoplasm and promotes EP300 dependent acetylation of ATG In the nucleus after starvation: BAG6 shuttles EP300 in the nucleus which leads to (i) decrease of ATGs proteins acetylation and (ii) P53 acetylation and expression of pro-autophagic ATG genes. Inhibition of autophagy: After its cleavage, BAG6 sequesters LC3-I leading to autophagy inhibition [44]

## 3. BAG Family Members in Mitophagy Regulation 

Mitochondria are complex organelles involved in many cellular processes, such as metabolism, energy production, apoptosis, calcium regulation, and different signaling pathways. Mitochondria are also the major source of reactive oxygen species. Due to their crucial role in cell homeostasis, the synthesis, degradation, and renewal of mitochondria must be tightly controlled. Mitochondria can be degraded by mitophagy [45]. One of the major signaling pathways involved in this process includes the kinase PINK1 and the E3 ubiquitin ligase Parkin. In basal conditions, PINK1 is processed by different proteases in the inner mitochondrial membrane and then relocates to the cytoplasm, where it is degraded by the proteasome. Upon cellular stress that leads to mitochondrial depolarization, PINK1 is stabilized and accumulates at the OMM, where it phosphorylates Parkin, promoting its localization to the OMM. Then, Parkin ubiquitinates mitochondrial proteins that are phosphorylated by PINK1, creating phospho-ubiquitin chains [46]. These chains are “eat me” signals recognized by specific mitophagy adaptors that harbor a LIR motif for interaction with LC3-II. Engulfment of damaged mitochondria is also ensured by receptors that harbor a LIR motif and are anchored to mitochondria (Figure 2).

Recent data show that BAG family members intervene in all the early steps of mitophagy, from regulating the mitochondrial morphology to the specific targeting of mitochondria to autophagomes (Table 2).

### 3.1. BAG Family Members and the Regulation of Mitochondrial Morphology

Mitochondrial morphology is dynamically regulated by fusion and fission events [47]. Recent studies described the role of BAG6, which is located in mitochondria, in mitochondrial morphology regulation. Due to size limitation, only fragmented mitochondria are engulfed into autophagomes [48]. In basal conditions, BAG6 is located in the mitochondrial matrix; however, after mitochondrial depolarization, it translocates to the OMM and induces mitochondrial fragmentation [49]. BAG6 also modulates mitochondrial morphology by interacting with the pro-fusion protein MNF2, thus promoting its proteasomal degradation in a cell model where expression of DRP1, a key regulator of fission, is downregulated [50]. These data suggest that BAG6 modulates the equilibrium between fusion and fission to maintain mitochondrial homeostasis. BAG6 also regulates the localization of mitochondria by controlling the cytoplasmic redistribution of depolarized mitochondria in the perinuclear region where mitophagy takes place [51]. Altogether, these findings suggest that BAG6 is a master regulator of mitophagy induction by controlling the morphology and cytoplasmic localization of mitochondria.

### 3.2. BAG Family Members and the Regulation of the PINK/Parkin Signaling Pathway

Depolarization of the mitochondrial membranes is an established mechanism for inducing mitophagy. It is mediated through PINK1 stabilization and localization at the OMM, followed by Parkin recruitment to mitochondria. The recognition of phospho-ubiquitinylated mitochondrial proteins by a mitophagic adaptor allows the engulfment of mitochondria in autophagosomes. Engulfment of damaged mitochondria is also ensured by receptors that harbor a LIR motif and are anchored to mitochondria (Figure 2). Interestingly, recent evidence indicates that BAG family proteins interact with PINK1 or PARKIN to modulate their activity. As mutations in PINK1 or PARKIN, two major mitophagy regulators, cause autosomal recessive PD, it is important to fully understand the role of BAG family members in their regulation.

#### 3.2.1. PINK1

BAG2 is an upstream regulator of the PINK1/Parkin signaling pathway. Indeed, BAG2’s direct interaction with PINK1 blocks PINK1 ubiquitination and degradation through the ubiquitin-proteasome pathway and promotes Parkin recruitment and then mitophagy [52,53]. It has been proposed that BAG2 expression decrease is an early-diagnosis plasma biomarker of PD [54]. Moreover, a mutant of PINK1, PINK1^R492X^, induces mitochondrial dysfunction and reactive oxygen species production. PINK1^R492X^ binds more tightly to BAG2 than wild type PINK1, suggesting an important role of BAG2 in PD neurodegeneration [52].

The molecule 1-methyl-4-phenylpyridinium (MPP^+^) is a neurotoxic molecule that interferes with oxidative phosphorylation in mitochondria by inhibiting complex I, leading to ATP depletion. In cells incubated with MPP^+^, BAG5 relocates to the mitochondria, interacts with PINK1, and decreases its ubiquitination, thus increasing its stability [55]. Similarly, reduction of BAG5 expression due to expression of miR-155, a miR expressed in aging and inflammation, destabilizes PINK1 and disrupts mitophagy in aged bone marrow tissues and in mesenchymal stem cells [56]. As observed for BAG1 and BAG3 (see Section 1), BAG5 function in mitophagy may be modified during aging.

Recently, we reported that BAG6 induces mitochondrial fragmentation and mitophagy by favoring PINK1/Parkin mitochondrial accumulation and the phospho-ubiquitination of mitochondrial proteins [49]. Chronic exposure to MPP^+^ of neuronal cells, which mimics PD, decreases PINK1 expression and enhances BAG6 expression. In the contest of PD, BAG6 interacts with PINK1, decreasing its stability. This suggests that BAG6 participates in PD pathogenesis by decreasing the endogenous PINK1 levels [57]. Conversely, BAG5 seems to protect against PD by compensating the loss of PINK1 after MPP^+^ incubation, thus preventing mitochondrial dysfunction [48].

#### 3.2.2. Parkin

BAG3 is a key regulator of Parkin activity both in physiological and pathological conditions. In neonatal rat ventricular cardiomyocytes, BAG3 downregulation by siRNA decreases Parkin expression and mitochondrial localization after incubation with carbonyl cyanide m-chlorophenyl hydrazine (CCCP), a mitochondrial uncoupling agent. This is followed by mitophagy impairment and accumulation of altered mitochondria [58]. This finding suggests that BAG3 is essential for Parkin-dependent mitophagy, probably through its mitochondrial relocalization after exposure to CCCP. In hereditary myofibrillary myopathies, BAG3 may be mutated on proline 209 (BAG3^P209L^). It has been proposed that autophagy and mitophagy machinery defects participate in the pathogenesis of this disease with deregulated P62, LC3, WIPI1, PINK1, and Parkin expression [59].

Surprisingly, BAG5 stimulates mitophagy through PINK1, but its interaction with Parkin leads to mitophagy inhibition. Specifically, BAG5 directly interacts with Parkin and inhibits its E3 ubiquitin ligase activity, leading to neuronal degeneration [60]. Another study reported that BAG5 has a dual role in the balance between cell death and survival. Indeed, BAG5 impairs mitophagy by suppressing Parkin recruitment to damaged mitochondria but enhances Parkin-mediated degradation of MCL-1 (a protein involved in mitophagy) and cell death after incubation with CCCP [61]. Therefore, BAG5’s role in PINK1/Parkin activity is still unclear and requires further investigations.

BAG4 acts as a negative regulator of Parkin through direct interaction that inhibits its translocation to depolarized mitochondria [62]. However, its role in mitophagy remains to be elucidated.

Lastly, we recently reported that BAG6 promotes PINK1 and Parkin recruitment to the mitochondrial membrane after incubation with CCCP, leading to the phospho-ubiquitination of mitochondrial proteins [49].

### 3.3. BAG Family Members as Mitophagy Receptors

The targeting of mitochondria to autophagosomes is ensured by cytoplasmic adaptors that bind to phospho-ubiquitinated OMM proteins or by receptors anchored to the mitochondrial membrane [63]. Receptors and adaptors bind to LC3-II via a LIR motif defined by the [W/F/Y]-x-x-[L/I/V] sequence [64].

For this review, we analyzed the sequences of BAG family members and found that human BAG3 (amino acids 33–38, 91–96, 145–150, 203–208) and human BAG6 (amino acids 159–164, 268–273, 274–279, 1016–1021) harbor four putative LIR motifs (https://ilir.warwick.ac.uk/kwresult.php, accessed on 1 November 2021). Moreover, a bioinformatic analysis reported that the LIR motifs of BAG3 are conserved in different species, but their role in selective autophagy remains to be elucidated [65].

We recently showed that BAG6 is detected in the mitochondrial matrix in basal conditions but translocates to the OMM after mitochondrial depolarization. Furthermore, BAG6 harbors putative LIR motifs, and, by site-directed mutagenesis, we demonstrated that the LIR motif at position 1016–1021 is essential for its interaction with LC3-II and mitophagy induction. This suggests that BAG6 is a mitophagy receptor [49]. Another study identified another putative LIR motif at position 132–135 (YVMV) in the BAG6 sequence. The authors showed that this LIR motif interacts preferentially with LC3-I and pro-LC3, leading to autophagy inhibition, probably by blocking LC3 lipidation when bound to BAG6 [44].

In addition, we cannot exclude that BAG family members may also modulate the activity of mitophagy receptors. For instance, in bovine urothelial cancer caused by papillomavirus infection, BAG3 is overexpressed and modulates mitophagy through interaction with the mitophagic receptors FUNDC1 [66], P62, BNIP3, and BNIP3L/NIX [67] and optineurin [68].

**Table 2:** Role of BAG family members in mitophagy regulation.

Bag Family Member	Role in Mitophagy
**BAG2**	Stimulates mitophagy [52,53]: Interacts with PINK1 and inhibits its degradation. Promotes Parkin recruitment to the mitochondria and mitophagy.
**BAG3**	Stimulates mitophagy: Recruited to mitochondria [59]. BAG3 silencing decreases Parkin expression and altered mitochondria are accumulated [59]. Interaction with the mitophagic receptor FUNDC1 [66], P62, BNIP3, NIX [67], Optoneurin [68]. The LIR motifs of BAG3 are conserved among species but their function is still unkown [65].
**BAG4**	Its role in mitophagy is unknown but BAG4 interacts with mitophagy regulators: Direct interaction with Parkin, inhibits its translocation to damaged mitochondria [62].
**BAG5**	Stimulates Mitophagy: Direct interaction with PINK1, increases its stability [55]. In aged bone marrow, the reduction of BAG5 destabilizes PINK1 and reduces mitophagy [56]. Inhibits mitophagy: Inhibits Parkin leading to dopaminergic neuron degeneration [60]. Direct interaction with Parkin and inhibition of its recruitment to the mitochondria leading to cell death after strong mitochondrial damages [61].
**BAG6**	Stimulates mitophagy: When localized in mitochondria, BAG6 promotes mitochondrial fission and PINK1/Parkin signaling [49]. Involved in the localization of mitochondria to the perinuclear region [51] New receptor for mitophagy [49]. Inhibits mitophagy in PD: Chronic MPP^+^ treatment increases the expression of BAG6 expression that interacts with PINK1 decreasing its stability [57].

## 4. Dual Role of BAG Family Members in the Regulation of Autophagy and Mitophagy: The Example of BAG6

BAG6 is implicated in autophagy and mitophagy at different steps of these processes and as a function of its intracellular localization. When located in the cytoplasm, BAG6 sequesters the acetyltransferase EP300, leading to ATG acetylation, a posttranslational modification known to inhibit autophagy [42,43]. During ER stress, the C-terminus of BAG6 is cleaved by caspase 3, and then BAG6 accumulates in the cytosol, where it binds to pro-LC3 or LC3-I via the LIR^132−135^ motif and suppresses autophagy, thus promoting apoptosis [44]. During starvation, BAG6 and EP300 are relocated to the nucleus, leading to ATG deacetylation and to EP300-dependent P53 acetylation and the expression of pro-autophagic genes. Thus, BAG6 nuclear localization allows autophagy induction.

In physiological conditions, BAG6 is also localized in the matrix of mitochondria. Interestingly, after mitochondrial depolarization, BAG6 translocates, by an unknown mechanism, to the OMM. There, BAG6 plays a key role in all mitophagic steps: (1) induction of mitochondrial fission; (2) activation of PINK1/Parkin signaling and stimulation of mitochondrial protein phospho-ubiquitination; and (3) induction of autophagy in a LIR-dependent manner, suggesting that BAG6 is a new mitophagy receptor. Indeed, mutation of the LIR^1016−1021^ motif suppresses BAG6 interaction with LC3-II, and consequently mitophagy is not induced after mitochondrial depolarization [49].

BAG6 acts in different autophagic processes and can stimulate autophagy or mitophagy, when needed. Moreover, it plays a role in all mitophagy steps. It can also interact with various autophagic effectors (LC3) and regulators (EP300, PINK1). It is now important to determine how BAG6 interacts with all of these proteins. For example, it cannot be excluded that these interactions affect BAG6 localization by hiding or unmasking its NLS motif. BAG6 appears to be a platform to which different proteins bind for the implementation of quality control in cells. During protein synthesis, the BAG6 NLS motif is masked when BAG6 binds to the cytoplasmic retention factor TRC35, forcing its cytosolic localization, where it interacts also with UBL4A. This complex allows the link between BAG6 and hydrophobic substrates at risk of aggregation to determine their fate: protection of the hydrophobic zone or proteasomal degradation [69].

Altogether, these data show that BAG6 is a hub that different partners can bind to, depending on the cell condition (e.g., stress) and its intracellular localization. Therefore, BAG6 is a master regulator of cell fate through its quality control function.

## 5. Conclusions

The role of the BAG family members in the regulation of autophagy and mitophagy is now well established; however, the underlying molecular mechanisms are largely unknown, thus raising many questions. The role of BAG members in various diseases, such as cancer, neurodegenerative disorders, and cardiomyopathies, needs to be thoroughly characterized.

Many studies reported the direct interaction of BAG members with the autophagic machinery. BAG2, BAG3, and BAG5 interact with P62. BAG1 and BAG6 can bind to LC3, and BAG6 acts as a mitophagy receptor. Interestingly, BAG family members can also interact with components of the signaling pathways involved in mitophagy. BAG3 interacts with many mitophagic receptors. BAG2 and BAG5 bind to PINK1 to inhibit its degradation and to allow mitophagy induction. BAG4 and BAG5 directly interact with Parkin, but this interaction inhibits Parkin activity and mitophagic function. Future studies should identify the domain(s) involved in BAG member interactions with autophagy and mitophagy regulators and the interplay among BAG members. In addition, BAG members modulate both autophagy and mitophagy, and it is important to precisely understand the underlying mechanisms. Due to their ability to bind to multiple proteins, BAG family members may act as molecular platforms for different autophagy/mitophagy regulators.

## Abbreviations

ATG	Autophagy-related proteins
BAG	BCL-2 associated athanogene
BCL-2	B-cell lymphoma 2
BCL2L13	BCL2 Like 13
BNIP3	BCL2-adenovirus E1B 19 kDa protein-interacting protein 3
BNIP3L	BCL2-adenovirus E1B 19 kDa protein-interacting protein 3-like
CASA	Chaperone-assisted selective autophagy
CCCP	Carbonyl cyanide m-chlorophenyl hydrazine
DRP1	Dynamin-related protein 1
ER	Endoplasmic reticulum
FKBP8	FK506-binding protein 8
HOPS	Homotypic fusion and protein sorting
HSC70	Heat shock cognate 71 kDa protein
HSP70	Heat shock protein 70 family
IMM	Inner mitochondrial membrane
I/R	Ischemia/reperfusion
(LAMP2a)	Lysosome-associated membrane glycoprotein 2a
LC3	Protein light chain 3
LIR	LC3 interacting region
MFN2	Mitofusin-2
MPP^+^	1-methyl-4-phenylpyridinium
NEF	Nucleotide exchange factor
NIX	Nip3-like protein X
OMM	Outer mitochondrial membrane
PI3KIII	Phosphatidylinositol 3-kinase class III
PINK1	PTEN-induced putative kinase 1
PLEKHM1	Pleckstrin homology domain-containing family member 1
PD	Parkinson’s disease
PE	Phosphatidyethanolamine
PHB2	Prohibitin 2
ROS	Reactive oxygen species
SNARE	Soluble NSF (N-ethylmaleimide-sensitive factor) attachment protein receptor
